# Acid-sensing ion channels in the vasculature: emerging roles in systemic and pulmonary circulations

**DOI:** 10.1007/s00424-026-03196-7

**Published:** 2026-07-13

**Authors:** Nikki L. Jernigan

**Affiliations:** Department of Cell Biology and Physiology, University of New Mexico School of Medicine, Albuquerque, NM USA

**Keywords:** Endothelium, Smooth muscle, Pulmonary hypertension, Store-operated calcium entry, Mitochondria dysfunction, Hypoxia

## Abstract

Acid-sensing ion channels (ASICs) are proton-gated members of the degenerin/epithelial sodium channel family that are emerging as multifaceted regulators of cardiovascular function. ASICs expressed in baroreceptor, cardiac, and skeletal muscle afferents contribute to reflex control of blood pressure, cardiac function, and sympathetic outflow. In vascular smooth muscle and endothelial cells, ASICs integrate mechanical, metabolic, and humoral signals to regulate vascular tone. In the systemic circulation, ASIC2 contributes to pressure-dependent vasoconstriction of renal and cerebral arteries, supporting blood flow autoregulation and protection against organ injury. In contrast, ASIC1a promotes vasodilation, contributing to nitric oxide-dependent dilation in the cerebral arteries and to endothelium-dependent hyperpolarization and vasodilation in mesenteric arteries. In the pulmonary vascular smooth muscle cells, ASIC1a plays a central role in acute hypoxic- and receptor-mediated vasoconstriction, a role that becomes increasingly important in chronic hypoxia-induced pulmonary hypertension. Under these conditions, metabolic reprogramming drives extracellular acidification and enhances ASIC1a trafficking to the plasma membrane, promoting sustained depolarization, augmented store-operated calcium entry, and a hyperproliferative, apoptosis-resistant smooth muscle phenotype. ASIC1a additionally regulates mitochondrial homeostasis by modulating mitochondrial membrane potential, redox balance, and apoptotic susceptibility. Chronic hypoxia redistributes ASIC1a from mitochondria to the plasma membrane, leading to mitochondrial dysfunction and cell survival signaling, key features of pulmonary vascular disease. This review summarizes current understanding of ASIC function in the systemic and pulmonary vasculature and highlights non-proton-mediated signaling mechanisms, emerging mitochondria-specific mechanisms, sex-related differences, and therapeutic opportunities and challenges in targeting ASIC-dependent signaling pathways in vascular disease.

The vascular system is a highly dynamic and coordinated network of arteries, veins, and capillaries, each composed of three distinct layers: the endothelium, the smooth muscle layer, and the adventitia. Blood vessels continuously adapt to mechanical, metabolic, and biochemical stimuli to maintain adequate tissue perfusion and overall homeostasis. In addition, cells within the blood vessel wall interact with neighboring cells in the local microenvironment, forming a vascular niche with fibroblasts, perivascular nerves, macrophages, adipocytes, pericytes, and stem/progenitor cells. The ability of cells to sense and integrate signals within the vascular niche depends on tightly regulated mechanisms. Among these, ion channels play a fundamental role in signal transduction. Dysregulation of ion channel expression or activity can therefore contribute directly to vascular dysfunction and disease.

Fluctuations in extracellular pH are a common feature of the vascular microenvironment. These pH shifts are not simply byproducts of pathological processes but serve as important signaling molecules that influence vascular homeostasis. As such, many cells sense and respond to changes in local proton concentrations. During metabolic stress, including ischemia, hypoxia, inflammation, and intense metabolic activity, acidic metabolites can accumulate, resulting in localized extracellular acidosis.

Acid-sensing ion channels (ASICs) are a family of proton-gated, voltage-insensitive cation channels within the degenerin/epithelial sodium channel (DEG/ENaC) superfamily. Initially characterized in the nervous system, ASICs have been widely implicated in sensory transduction, nociception, and neuronal injury (most recently reviewed in [1]). More recently, ASICs have been identified in non-neuronal tissues, including cardiac myocytes, fibroblasts, vascular smooth muscle cells, and endothelial cells [2–5], indicating broader physiological roles beyond the nervous system.

The implication of ASICs in several vascular pathologies, including ischemia-reperfusion injury, hypertension, atherosclerosis, and pulmonary vascular disease, suggests that they play important roles in cardiovascular regulation. In this review, we first discuss ASIC expression and function in sensory afferents and their contribution to reflex control of blood pressure, cardiac function, and sympathetic outflow. We then focus on ASIC expression and function in vascular smooth muscle and endothelial cells, describing how ASIC-mediated cation conduction influences vascular tone, endothelial signaling, mitochondrial function, and vascular remodeling. We further discuss how pathological activation of ASICs contributes to vascular dysfunction and evaluate their potential as therapeutic targets in vascular disease.

## Molecular structure and classification of ASICs

Four ASIC genes (ACCN1-4) encode at least six distinct ASIC subunits, including ASIC1a, ASIC1b, ASIC2a, ASIC2b, ASIC3, and ASIC4 [6–11]. Each subunit consists of two hydrophobic transmembrane regions, short intracellular N- and C-termini, and a large extracellular loop that mediates proton sensing and mechanotransduction. ASIC1, ASIC2, and ASIC3 subunits assemble as homomeric or heteromeric trimers to form voltage-independent, proton-gated cation channels. While most ASICs are primarily permeable to Na^+^, homomeric ASIC1a and heteromeric ASIC1a/2b also permit Ca^2+^ influx [10, 12, 13]. The resulting cation entry contributes to membrane depolarization and activation of Ca^2+^-dependent signaling pathways. ASIC4, although detected in sensory neurons and the vasculature, does not form functional proton-gated channels. Instead, it has been shown to act as a modulator of other ASIC subunits in neurons [7, 14]. However, whether ASIC4 plays a similar regulatory role in vascular ASIC complexes remains an open question.

ASIC subunits exhibit distinct tissue distributions, biophysical properties, and pharmacological sensitivities, such that subunit composition within a trimeric complex is a primary determinant of channel function. This heterogeneity gives rise to channels with varying pH sensitivity, gating kinetics, and ion permeability [15–19]. For example, ASIC1a and ASIC3 are among the most pH-sensitive isoforms, with activation thresholds near physiological pH (pH ~ 7.0), whereas ASIC2 channels typically require more severe acidosis (pH < 5) for activation [1]. Although ASICs are classically activated by extracellular acidosis, their activity is also modulated by a variety of non-proton ligands, effector proteins, and signaling molecules [20, 21], indicating that complex regulatory mechanisms fine-tune channel gating and downstream signaling.

## ASICs in cardiovascular homeostasis

Cardiovascular homeostasis is essential for maintaining blood perfusion and tissue oxygenation. Changes in arterial blood pressure, blood volume, pH, oxygen, carbon dioxide, or metabolites are sensed by afferent neurons in the petrosal, nodose, and dorsal root ganglia (DRG), which relay these signals to the brainstem and spinal cord, thereby mediating reflex control of blood pressure and blood flow. ASIC1, ASIC2, ASIC3, and ASIC4 are expressed in DRG neurons [22, 23], with subunit-specific distribution across neuronal populations, suggesting that each isoform contributes to distinct sensory modalities involved in cardiovascular reflex control, particularly under metabolic stress.

### ASICs in baroreceptors and carotid body chemoreceptors

Baroreceptors are mechanoreceptors located in the walls of the aortic arch and carotid sinuses that detect changes in blood pressure and elicit negative feedback to stabilize it. ASIC1a, −1b, −2a, −2b, and − 3 are expressed in the nodose ganglia innervating these arterial baroreceptors, with ASIC2b being the predominate subunit [24]. Deletion of ASIC2 in null mice decreased baroreceptor sensitivity and vagal tone, resulting in increased sympathetic activity, hypertension, and tachycardia, demonstrating that ASIC2 is required for normal arterial baroreceptor mechanosensation [24].

Carotid bodies are chemoreceptors located at the bifurcation of the carotid arteries that detect changes in arterial pH, oxygen, and carbon dioxide. Acidosis, hypoxia, and hypercapnia elicit reflex increases in ventilation and sympathetic nerve activity, raising heart rate and blood pressure. ASIC1b and ASIC3 are more prominently expressed in carotid bodies than ASIC2 [25], and ASIC3 has been implicated as a key mediator of acid sensing in peripheral chemoreceptors [25–28]. Consistent with these findings, ASIC3 null mice exhibit an imbalance in autonomic regulation, with decreased sympathetic function and lower blood pressure [29, 30].

### Cardiac ischemic sensing

The heart receives dual afferent innervation from vagal fibers, with cell bodies in the nodose ganglia, and sympathetic fibers, with cell bodies in the DRG. ASICs are expressed in both pathways but appear to serve distinct functional roles: mechanosensors in vagal nodose afferents and chemosensors in sympathetic afferents. Native acid-evoked currents in cardiac DRG neurons share functional properties of ASIC2a/3 heteromeric channels, including their rapid gating kinetics and high pH sensitivity, implicating these subunits as molecular sensors of cardiac ischemia and angina [31–35]. In addition to their chemosensory role, ASIC3 functions as a mechanosensor at the venoatrial junction, where it contributes to blood volume regulation by modulating reflex-mediated release of atrial natriuretic peptide and sympathoinhibition [36].

ASICs are also expressed within the myocardium itself. ASIC1 and ASIC3 are highly expressed in the human left ventricle and have been identified in cardiomyocytes, endothelial cells, and fibroblasts of the mouse heart [4]. Consistent with a role in ischemic injury, genetic and/or pharmacological inhibition of ASIC1a and ASIC3 improves cardiomyocyte viability following acute ischemia-reperfusion injury [4, 37]. However, this protective effect of ASIC3 inhibition contrasts with findings from ASIC3 null mice, which show prolonged ST-segment depression and severe cardiac fibrosis following isoproterenol-induced ischemia [38], as well as disruptions in cardiac autonomic balance [29, 30]. These seemingly divergent findings may reflect distinct roles for ASIC3 in acute cardiomyocyte injury versus chronic autonomic and structural remodeling, highlighting context-dependent functions of ASIC channels in the heart.

### Skeletal muscle exercise pressor reflex

The exercise pressor reflex is a critical autonomic feedback mechanism that increases arterial pressure, heart rate, and ventilation during physical activity, ensuring adequate perfusion of active skeletal muscle. Mechanosensitive and metabosensitive skeletal muscle afferents respond to both muscle contraction and the accumulation of metabolic byproducts. ASIC1a, ASIC2, and ASIC3 are expressed in afferent endings innervating skeletal muscle and contribute to the metabolic component of the exercise pressor reflex [39–43].

In heart failure, exercise intolerance is associated with an exaggerated exercise pressor reflex. This response has been linked to a shift in ASIC subunit composition within muscle afferents, from ASIC1a/2a/3 and ASIC1a/3-containing channels towards ASIC2a/3 heteromers. This shift alters channel pH sensitivity, making afferent neurons more responsive to exercise-induced acidosis and likely contributing to the exaggerated sympathetic response observed in heart failure [44]. ASIC1a has also recently been implicated in the mechanical component of exercise pressor reflex in animals with heart failure [45]. Consistent with a role in exercise pressor reflex, ASIC1a null mice show enhanced functional hyperemia and increased peak exercise capacity [46]. However, rather than the enhanced vasodilatory capacity of individual vessels, this effect appears to result from increased vascular recruitment within the hind-limb skeletal muscle bed [46].

### ASICs and blood pressure regulation

Together, these findings suggest that ASICs contribute to the regulation of acute cardiovascular reflexes, with potential downstream consequences for long-term blood pressure regulation. Consistent with the mechanosensory role of ASIC2 in baroreceptor reflex, ASIC2 null mice exhibit elevated blood pressure [24]. In contrast, ASIC3 null mice display reduced blood pressure, consistent with loss of chemosensitive sympathoexcitatory drive [29]. Global deletion of ASIC1a does not significantly affect blood pressure in young adult mice [46, 47]. Likewise, cell-specific deletion of ASIC1a in vascular smooth muscle cells (VSMCs) or endothelial cells has no significant impact on blood pressure [3], suggesting that ASIC1a plays a minimal role in blood pressure regulation under basal physiological conditions. Nevertheless, growing evidence indicates that ASIC1a may assume greater importance in pathological states.

In support of this notion, genetic deletion of ASIC1a results in age-dependent hypertension selectively in male mice, while female ASIC1a null mice are protected from age-related hypertension [47]. In males, this hypertensive phenotype is associated with increased sympathetic nerve activity, hyperaldosteronism, and increased corticosterone levels, but occurs independently of renin-angiotensin system activation. Additionally, male ASIC1a null mice show reduced sensitivity to angiotensin II-induced hypertension [47]. These findings suggest that ASIC1a contributes to cardiovascular regulation through sex-dependent neuroendocrine mechanisms, with direct hormonal regulation of ASIC1a representing a potential mechanism for this difference. β-estradiol has been shown to promote ASIC1a protein degradation in cortical neurons and articular chondrocytes, protecting against acidosis-mediated neurotoxicity [48, 49]; conversely, β-estradiol potentiates ASIC1a activity in sensory neurons [50]. These tissue-dependent, opposing effects of estrogen on ASIC1a suggest that hormonal regulation of ASIC1a expression or function is plausible, though it remains to be determined whether estrogen similarly modulates ASIC1a in vascular or neuroendocrine tissues relevant to blood pressure regulation.

Together, these observations establish ASICs as important modulators of cardiovascular homeostasis via reflex pathways that regulate blood pressure and volume. However, ASIC function is not limited to these integrated neural reflex pathways. ASICs are also directly expressed within the vascular wall itself, where both their expression patterns and functional roles differ between the systemic and pulmonary circulations.

## ASICs in the systemic vasculature

In addition to their roles in systemic blood pressure regulation, ASICs are increasingly recognized as direct regulators of vascular reactivity, the dynamic process by which vascular smooth muscle cells (VSMCs) contract and relax to adjust vascular resistance and match blood flow with metabolic demand. Vascular tone refers to the degree of constriction of a blood vessel at a given time relative to its maximally dilated state. Most blood vessels have a basal tone that is influenced by multiple interacting mechanisms, including sympathetic nerve activity, circulating hormones, endothelial-derived factors, mechanical forces, and local metabolic signals. Increases in intracellular Ca^2+^ ([Ca^2+^]_*i*_) play a central role in the excitation, contraction, transcription, and proliferation of VSMCs. In VSMCs, Ca^2+^ entry occurs through a combination of voltage-gated Ca^2+^ channels and a variety of non-voltage-gated cation channels, including mechanosensitive and ligand-gated channels. Increases in [Ca^2+^]_*i*_ are also critical for the release of endothelial-derived factors that act on underlying VSMCs. Endothelial cells are considered non-excitable, and Ca^2+^ influx occurs mainly through non-voltage-gated cation channels [51, 52]. These channels are also the primary source of Na^+^ entry into VSMCs and endothelial cells, as voltage-gated Na^+^ channels are largely absent from the vasculature [53].

Among the non-voltage-gated cation channels expressed in the vasculature, four major activation mechanisms predominate: mechanosensitive, ligand-gated, receptor-operated, and store-operated. Receptor-operated and store-operated channels are typically activated downstream of Gq protein-coupled receptor signaling, in which phospholipase C generates inositol 1,4,5-trisphosphate (IP₃) and diacylglycerol. IP_3_ promotes Ca^2+^ release from the endoplasmic reticulum (ER), or sarcoplasmic reticulum (SR) in smooth muscle, with subsequent store depletion triggering store-operated Ca^2+^ entry across the plasma membrane. Concurrently, diacylglycerol activates receptor-operated Ca^2+^ entry either by directly binding the channel or via protein kinase C (PKC). Additionally, mechanosensitive ion channels respond directly to changes in blood pressure and shear stress, contribution to myogenic tone and flow-mediated vascular responses, respectively. Because ASICs are non-voltage-gated cation channels permeable to both Na^+^ and Ca^2+^, and have been implicated in both ligand-gated and mechanosensitive signaling in other tissues, they may represent an important yet underappreciated mechanism in directly regulating vascular reactivity.

The functional impact of ASIC activity in the vascular wall largely depends on the cell type in which it is expressed. In VSMCs, increases in [Ca^2+^]_*i*_ or membrane depolarization promote contraction, whereas in endothelial cells, elevations in [Ca^2+^]_*i*_ or membrane hyperpolarization stimulate the release of vasodilatory factors. Thus, ASIC activation may have different effects on vascular tone depending on the cellular context. ASIC1, ASIC2, and ASIC3 isoforms have been reported in both VSMCs and endothelial cells [5, 54–60], though their functional roles vary considerably across vascular beds. The following sections examine ASIC function across specific vascular beds to further define their region- and cell type-dependent roles in vascular regulation.

### Cultured vascular smooth muscle cell model

Early evidence for such context specificity came from studies in A10 cells, a vascular smooth muscle-like cell line derived from the embryonic rat thoracic aorta. ASIC1, ASIC2, and ASIC3 are expressed in these cells and contribute to wound healing responses [58]. In contrast, chemotactic migration in response to platelet-derived growth factor-BB is differentially regulated by ASIC isoforms: siRNA knockdown of ASIC1 or ASIC3 attenuates migration, whereas ASIC2 knockdown increases this response [58]. Together, these data provide evidence that individual ASIC subunits can differentially regulate VSMC behavior, leading to distinct and sometimes opposing functional outcomes.

### Cutaneous vasculature

Cutaneous pressure-induced vasodilation is a protective physiological response in which direct, low-level pressure on the skin triggers localized increases in blood flow. This mechanism results from cutaneous neuro-vascular interaction and prevents pressure-related tissue ischemia. Pressure-induced vasodilation is impaired by conditions that damage sensory nerves or the microvascular endothelium, such as aging, diabetes, and peripheral neuropathy, and is directly linked to an increased risk of microvascular dysfunction and skin lesions, including pressure ulcers. ASIC3 is an essential neuronal sensor of cutaneous pressure in both humans and rodents and is required for protection against pressure ulcers in mice [61]. ASIC3 activation triggers the release of the vasodilatory neuropeptide CGRP from sensory nerve terminals, which acts directly on VSMCs to promote vasodilation and increase local cutaneous blood flow. Together, these findings establish a neurogenic mechanism by which ASIC3-mediated sensory transduction couples mechanical stimuli to local vasoprotective responses in the skin.

### Cerebral vasculature

In the cerebral vasculature, expression of multiple ASIC isoforms, including ASIC1a, ASIC1b, ASIC2a, ASIC2b, ASIC3, and ASIC4, have been detected [54, 55, 60]. In freshly isolated cerebral VSMCs, robust expression of ASIC1b and ASIC2a has been reported, with comparatively weaker expression of ASIC1a and ASIC3. Consistent with this expression profile, cerebral VSMCs exhibit proton-evoked ASIC-like currents that appear to be mediated predominantly by ASIC1b [55].

**Fig. 1:**
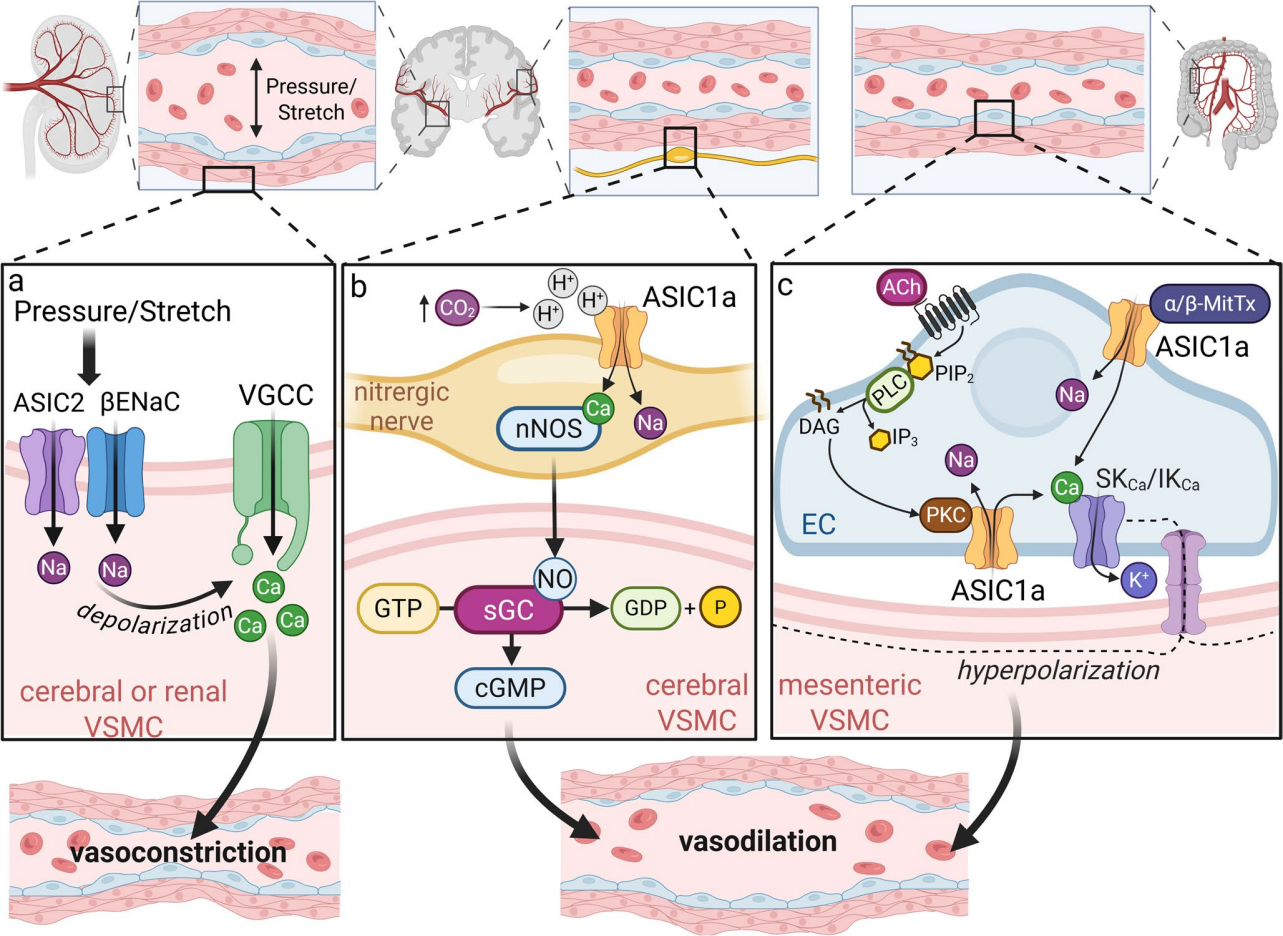
Differential roles of ASICs in systemic vascular function. (**a**) In renal afferent and middle cerebral arteries, ASIC2, together with βENaC, function as a mechanotransducer of the myogenic response. Increased arterial pressure causes Na^+^ influx, leading to VSMC depolarization, activation of voltage-gated Ca^2+^ channels (VGCC), and vasoconstriction. This autoregulatory mechanism is essential for maintaining tissue perfusion and protecting against organ injury. (**b**) In cerebral pial arteries, hypercapnia activates neuronal ASIC1a, thereby stimulating the Ca^2+^-sensitive neuronal nitric oxide synthase (nNOS). Nitric oxide (NO) diffuses to the adjacent cerebral VSMCs, where it activates soluble guanylyl cyclase (sGC), increasing cyclic GMP (cGMP) signaling and promoting vasodilation. (**c**) In mesenteric endothelial cells (EC), acetylcholine binds muscarinic receptors coupled to phospholipase C (PLC), resulting in hydrolysis of phosphatidylinositol 4,5-bisphosphate (PIP_2_) into diacylglycerol (DAG) and inositol 1,4,5-trisphosphate (IP_3_). ASIC1a is activated primarily through receptor-operated mechanisms involving DAG/protein kinase C (PKC) signaling and can also be directly activated by the selective agonist, α/β-MitTx. ASIC1a-mediated Ca^2+^ influx within endothelial microdomains activates small- and intermediate-conductance Ca^2+^ activated K^+^ channels (SK_Ca_/IK_Ca_), producing endothelial hyperpolarization that is transmitted to adjacent VSMCs through myoendothelial gap junctions, resulting in vasodilation. Together, ASIC1a-dependent vasodilation in cerebral and mesenteric arteries increases blood flow during periods of increased metabolic demand and facilitates the clearance of metabolic byproducts. Figure created with BioRender.com

Support for a role of ASICs in regulating cerebrovascular tone comes from both pharmacologic and genetic studies. Pharmacological inhibition of ASICs with amiloride enhances lactate-induced relaxation in rat middle cerebral arterial rings [54], whereas genetic deletion of ASIC2 reduces basal vascular tone without affecting vasoconstrictor responses to membrane depolarization or phenylephrine [62]. Although these results indicate that ASICs contribute to the maintenance of basal cerebrovascular tone, pressure-dependent myogenic responses are preserved in ASIC2-null mice. In contrast, myogenic responses are abolished in ASIC2-heterozygous mice [62], highlighting a more complex role for ASIC2 in cerebrovascular function. This divergent phenotype has been attributed to compensatory upregulation of the related γ-ENaC subunit in ASIC2-null mice, whereas reduced expression of γ-ENaC and β-ENaC in ASIC2-heterozygous mice may disrupt mechanosensitive signaling [62], indicating that both ASIC2 and β-ENaC are required for pressure-induced tone (Fig. 1a). Supporting an intrinsic mechanosensory capacity for such complexes, both ASIC homomers and ASIC2a/βENaC heteromeric channels expressed in *Xenopus oocytes* are directly activated by shear stress [63, 64], though whether this extends to flow-mediated vasodilation remains to be established. These observations align with the established role of ENaC subunits in mediating pressure-induced mechanotransduction in VSMCs, as described by Drummond and colleagues [65].

In contrast to these mechanisms, ASIC1a has been implicated in hypercapnia-induced vasodilation in cerebral arteries [66, 67] (Fig. 1b). This effect appears to be mediated predominantly by neuronal ASIC1a, as neuron-specific deletion of ASIC1a similarly attenuates hypercapnia-evoked, nitric oxide-mediated vasodilation. In contrast, acetylcholine-induced, endothelium-dependent cerebral vasodilation is unaffected by either global ASIC1a deletion or pharmacological inhibition with psalmotoxin-1 [66], indicating that ASIC1a does not directly regulate endothelial nitric oxide synthase-dependent signaling pathways in the cerebral vasculature.

### Renal vasculature

ASIC2 plays a central role in renal autoregulation. In renal interlobar arteries, pressure-induced constriction is impaired in both ASIC2-heterozygous and ASIC2-null mice [68]. The physiological significance of this impaired mechanotransduction has been demonstrated in vivo, where restoration of renal blood flow in response to a stepwise increase in perfusion pressure is blunted in ASIC2-null mice, with more pronounced deficits observed in ASIC2-heterozygous mice [68]. Functionally, these alterations are accompanied by mild renal injury and elevated blood pressure, closely resembling phenotypes reported in mice with reduced expression of β-ENaC [69, 70]. Consistent with this parallel, Lu and colleagues demonstrated that ASIC2 associates with β-ENaC in VSMCs to mediate pressure-induced constriction in renal afferent arterioles [71]. These data suggest that ASIC2-βENaC signaling complexes are fundamentally important to renal vascular mechanotransduction (Fig. 1a), as disruption of either component compromises renal autoregulation and is associated with renal injury and hypertension.

### Mesenteric vasculature

ASIC1a is expressed in both VSMCs and endothelial cells of mesenteric resistance arteries. Although expressed in VSMCs, ASIC1a does not appear to directly contribute to receptor-mediated vasoconstriction in this vascular bed [2, 72]. In contrast, inhibition of ASIC1a enhances vasoconstrictor responsiveness to low concentrations of endothelin-1, suggesting an opposing, vasodilatory influence. Because endothelin-1 receptors are expressed on both VSMCs (ET_A_ and ET_B_ receptors) and endothelial cells (ET_B_ receptors), mediating vasoconstriction and vasodilation, respectively [73], this data implies that endothelial ASIC1a counteracts vasoconstrictor signaling. Consistent with this interpretation, inhibition of ASIC1a with psalmotoxin-1 or genetic deletion of ASIC1a increases basal tone, elevates VSMC [Ca^2+^]_*i*_, and attenuates endothelium-dependent vasodilation in small mesenteric arteries [2].

#### Mechanisms of ASIC1a activation in mesenteric endothelial cells

Mechanistically, ASIC1a promotes endothelium-dependent vasodilation by mediating Ca^2+^ influx into the endothelial cells. Inhibition of ASIC1a markedly reduces endothelial Ca^2+^ responses and impairs vasodilation in response to acetylcholine [2]. Acetylcholine activates muscarinic type 3 receptors on endothelial cells, which are Gq protein-coupled receptors that increase [Ca^2+^]_*i*_ through ER Ca^2+^ release, store-operated Ca^2+^ entry, and receptor-operated Ca^2+^ entry. In mesenteric endothelial cells, however, ASIC1a-dependent Ca²⁺ influx occurs predominantly via receptor-operated rather than store-operated mechanisms, and likely involves PKC signaling [2] (Fig. 1c). These findings are consistent with evidence that PKC regulates ASIC1a activity across multiple systems [74–77], linking receptor activation to ASIC1a function in the endothelium.

#### Downstream signaling and microdomain localization in mesenteric endothelial cells

Increases in endothelial [Ca^2+^]_*i*_ stimulate the production and release of endogenous vasodilators, including nitric oxide and prostacyclin, and activate endothelium-dependent hyperpolarization (EDH). In small mesenteric resistance arteries, EDH is the dominant vasodilatory mechanism [78], and ASIC1a plays a key role in this pathway [2]. EDH is initiated when local elevations in endothelial [Ca^2+^]_*i*_ activate intermediate- and small-conductance Ca^2+^-activated K^+^ channels (IK_Ca_ and SK_Ca_), resulting in hyperpolarization. This hyperpolarizing current spreads to adjacent VSMCs through myoendothelial gap junctions, promoting VSMC relaxation (Fig. 1c).

The functional contribution of ASIC1a also depends on its subcellular localization. In mesenteric resistance arteries, ASIC1a is enriched at myoendothelial junctions, where it colocalizes with IK_Ca_ and SK_Ca_ channels to mediate EDH [2]. Consistent with this spatial organization, selective activation of ASIC1a using α/β-MitTx enhances IK_Ca_/SK_Ca_ channel current and induces vasodilation, providing direct evidence that ASIC1a functions upstream of EDH signaling. Similar mechanisms have been described in neurons, where activation of ASIC1a disrupts inhibitory coupling to large-conductance Ca^2+^-activated K^+^ (BK_Ca_) channels, thereby enhancing their activity [79, 80].

These findings contrast with those in the cerebral circulation, where acetylcholine-induced vasodilation is unaffected by ASIC1a inhibition [66]. In general, EDH contributes more to vasodilation in smaller vessels than in larger conduit arteries; however, its contribution also varies substantially across vascular beds [81–83]. Faraci and colleagues demonstrated that acetylcholine-induced vasodilation in cerebral pial arterioles is predominantly nitric oxide-dependent [66], whereas more than 65% of the vasodilatory response in small mesenteric arteries is independent of nitric oxide and cyclooxygenase pathways [2, 84–86]. Importantly, this EDH-mediated response is abolished by inhibition of either IK_Ca_/SK_Ca_ channels or ASIC1a [2], indicating convergence on a shared signaling axis.

### Physiologic and pathologic importance of ASIC in systemic vascular function

Collectively, the findings described above and summarized in Fig. 1 demonstrate that ASICs differentially regulate systemic vascular function across vascular beds and contribute to the control of vascular tone, blood flow, and tissue perfusion. The contribution of ASIC2 to vascular mechanotransduction and blood flow regulation in renal and cerebral arteries is an essential function for maintaining tissue perfusion and protecting against organ injury. In contrast, ASIC1a mediates vasodilation in cerebral and mesenteric arteries, thereby augmenting blood flow, serving as a critical response to increased metabolic demand, and facilitating the clearance of metabolic byproducts.

The broader contribution of ASICs to systemic vascular regulation remains incompletely defined. Whether ASIC-dependent signaling extends to additional vascular beds or involves other cell types within the vascular niche remains an important area for investigation. ASIC1, ASIC2, and ASIC3 have been detected in various inflammatory cells, including macrophages, dendritic cells, central microglia, Th cells, eosinophils, neutrophils, mast cells, B cells, plasma cells, and T cells, where they have been implicated in processes such as phagocytosis, maturation, and cytokine production [87–94], suggesting immune-cell ASICs may indirectly influence vascular function by modulating endothelial activation, vascular permeability, leukocyte recruitment, and vascular remodeling in inflammatory diseases. Consistent with this concept, recent studies demonstrate that macrophage ASIC1a promotes atherogenesis [95], while ASIC3 drives macrophage polarization and fibroblast-to-myofibroblast differentiation, a key process in fibrosis and vascular remodeling [96]. Collectively, these findings suggest that ASICs function as sensors of tissue acidosis, coupling extracellular pH changes to immune and inflammatory responses and potentially contributing to the pathogenesis of atherosclerosis, hypertension, ischemia-reperfusion injury, and other vascular diseases. Further studies will be required to define the extent to which these immune-mediated actions contribute to the physiological and pathological regulation of systemic vasculature.

## ASICs in the pulmonary vasculature

Unlike the systemic circulation, the pulmonary arteries are thin-walled, carry deoxygenated blood, and constrict rather than dilate in response to hypoxia. ASIC1, ASIC2, and ASIC3 are expressed in pulmonary arterial VSMCs and endothelial cells [56, 59]. Functional studies using isolated, saline-perfused lungs indicate that ASIC1a does not substantially contribute to endothelial-dependent pulmonary vasodilation but instead plays a key function in mediating pulmonary vasoconstriction, including responses to acute alveolar hypoxia and receptor-mediated agonists [97]. In line with these observations, ASIC1a promotes increases in VSMC [Ca^2+^]_*i*,_ in part through activation of store-operated Ca^2+^ entry pathways [59, 97, 98], implicating ASIC1a as an important contributor to pulmonary vasoconstrictor signaling.

In contrast, ASIC2 acts as a counter-regulatory influence in the pulmonary circulation. Mice lacking ASIC2 exhibit increased baseline pulmonary vascular resistance and enhanced vasoconstrictor responses to hypoxia, depolarizing stimuli, and receptor-mediated agonists [56]. This heightened reactivity is associated with increased basal tone, elevated VSMC [Ca^2+^]_*i*_, and enhanced store-operated Ca^2+^ entry. These effects occur independently of L-type voltage-gated Ca^2+^ channel activation and are abolished by inhibition of ASIC1a. Furthermore, it appears that this ASIC2-dependent restraint of ASIC1-dependent Ca^2+^ signaling occurs through functional mechanisms, rather than changes in ASIC1a expression [56]. One possible explanation is that ASIC2 co-assembles with ASIC1a to form heteromeric channels with distinct biophysical properties that limit ASIC1a activity. While evidence for βENaC/ASIC2 complexes exists in VSMCs [71], direct evidence for ASIC1a/ASIC2 or ASIC2/ASIC3 heteromerization in vascular cells is currently lacking, and it remains an important open question whether such heteromers contribute to the counter-regulatory influence of ASIC2 on pulmonary vascular reactivity.

ASIC3 appears to play a more limited role in pulmonary vascular regulation. ASIC3-null mice show increased hypoxic- and receptor-mediated vasoconstriction without corresponding changes in basal tone or VSMC [Ca^2+^]_*i*_ [56]. Although the underlying mechanism remains unresolved, the established role of ASIC3 in peripheral chemoreceptor reflex [24–26, 28] may indirectly affect pulmonary vasoreactivity. Using whole-body plethysmography, studies show that baseline minute ventilation and responses to hypercapnia are preserved across ASIC1-, ASIC2-, and ASIC3-null mice [99]. While ASIC2-null mice exhibit an attenuated ventilatory response to acute isocapnic hypoxia, studies in chronically hypoxic ASIC2-null mice exhibit increased minute ventilation driven by greater tidal volume, with no alterations in arterial blood gases or pH [56, 99]. These data argue against worsened hypoxemia as the primary cause of the exacerbated pulmonary vasoconstriction observed in ASIC2- and ASIC3-null mice and enhanced pulmonary hypertension in ASIC2-null mice, and instead support a dominant role for local, intrinsic pulmonary vascular mechanisms.

### Store-dependent mechanisms of ASIC1a activation in pulmonary VSMCs

**Fig. 2:**
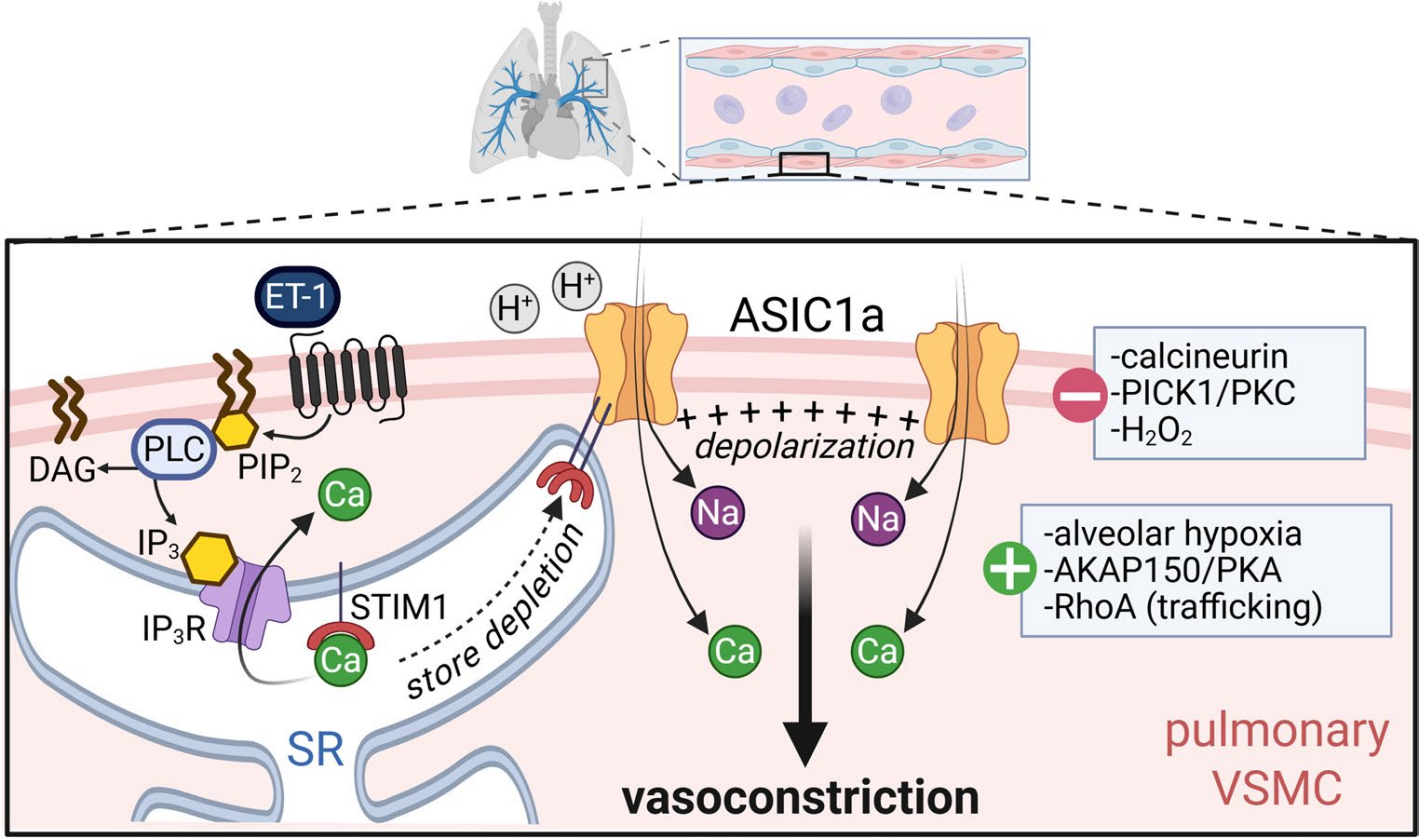
Activation and Regulation of ASIC1a in Pulmonary Vascular Smooth Muscle Cells. Pulmonary arteries are thin-walled and carry deoxygenated blood. Agonist binding to Gq-protein-coupled receptors, such as endothelin-1 (ET-1), promotes IP_3_ Ca^2+^ release from the sarcoplasmic reticulum (SR). Depletion of SR Ca^2+^ is sensed by stromal interaction molecule 1 (STIM1), which oligomerizes and translocates to the SR-plasma membrane junctions where it interacts with and activates ASIC1a. ASIC1a activation results in Na^+^ and Ca^2+^ influx, contributing to membrane depolarization and vasoconstriction. In addition to traditional H^+^, ASIC1a is also activated following acute alveolar hypoxia. ASIC1a activity in pulmonary VSMC is positively modulated by PKA and RhoA (primarily through plasma membrane trafficking); whereas calcineurin, PICK1/PKC, and hydrogen peroxide (H_2_O_2_) negatively regulate plasma membrane channel activity. Figure created with BioRender.com

In contrast to mesenteric endothelial cells, where ASIC1a-mediated Ca^2+^ influx occurs predominantly through receptor-operated, PKC-dependent mechanisms that promote endothelial hyperpolarization and vasodilation, ASIC1a in pulmonary VSMCs is functionally linked to store-operated Ca^2+^ entry pathways and vasoconstriction, and PKC signaling suppresses rather than potentiates ASIC1a activity [59, 72, 75, 97, 98] (Fig. 2). This divergence may be explained, at least in part, by differential PKC isoform expression or engagement of distinct phosphorylation sites across vascular beds. Notably, PKCβI and PKCβII are functionally expressed in pulmonary but not mesenteric arteries [100], and PKCβI/II has been shown to inhibit ASIC1 inward current in glioma cells [74]. This could account for PKC-mediated inhibition of ASIC1a in pulmonary vs. mesenteric arteries. Alternatively, at the molecular level, two PKC consensus phosphorylation sites, S40 and S499, have been identified on human ASIC1b that differentially mediate PKC-dependent activation versus inhibition when expressed in *Xenopus oocytes* [101], illustrating how site-specific phosphorylation can produce divergent functional outcomes depending on cellular context.

Consistent with a store-operated Ca^2+^ entry mechanism, ASIC1a is localized in intracellular compartments, including the ER, and can rapidly translocate to the plasma membrane in response to stressors such as serum or insulin deprivation in neurons [102]. Similar requirements for Ca^2+^ release and store depletion have been described for related DEG/ENaC family members, including *mec-4(d)* in *Caenorhabditis elegans* [103, 104]. Further supporting a role for ASIC1a in store-operated Ca^2+^ entry, ASIC1a has been shown to interact with stromal interaction molecule 1 (STIM1) [72], the ER Ca^2+^ sensor that couples store depletion to activation of Ca^2+^-permeable channels at the plasma membrane [105, 106] (Fig. 2). ASIC1a also exhibits appreciable Ca^2+^ permeability in VSMCs, with Na^+^/Ca^2+^ permeability ratios of approximately 2.0 in freshly isolated cells and ~ 2.5 in primary cultured VSMCs [59]. While some studies report higher Na^+^/Ca^2+^ ratios of ~ 10, suggesting lower Ca^2+^ permeability [1], these discrepancies likely reflect differences in species, cell type, expression system, stimulus, recording conditions, and Ca^2+^ concentration, since extracellular Ca^2+^ is known to inhibit ASIC currents and influence permeability ratios [59, 107]. The Ca^2+^ permeability values reported in VSMCs are consistent with earlier measurements in *Xenopus* oocytes (~ 2.5) [10] and functional evidence for Ca^2+^ entry through ASIC1a in native vascular cells. Furthermore, ASIC1a-dependent Ca^2+^ influx occurs independently of L-type voltage-gated Ca^2+^ channel activity, supporting its role in Ca^2+^ entry following store depletion [59, 97, 98].

ASIC channels are classically characterized by transient activation in response to acute pH changes, which would limit their contribution to sustained vascular tone under steady-state conditions. However, ASIC1a exhibits stimulus-dependent gating behavior in pulmonary VSMCs, in which store depletion induces a more sustained current, distinct from the transient response to acidosis [59], suggesting distinct modes of channel regulation. Furthermore, under basal physiological conditions, the contribution of ASIC1a to store-operated and agonist-mediated pulmonary vasoconstriction is minimal; however, its functional importance is markedly enhanced following chronic hypoxia, which coincides with increased ASIC1a surface expression in VSMCs [108].

### Acute hypoxia-dependent ASIC1a activation

One of the primary physiological stimuli for pulmonary vasoconstriction is alveolar hypoxia. This hypoxic pulmonary vasoconstriction redirects blood flow to better-ventilated regions of the lung, thereby matching perfusion to ventilation and optimizing arterial oxygenation. Although mitochondria are widely accepted as oxygen sensors in pulmonary VSMCs, and electron transport chain-derived reactive oxygen species have been implicated in downstream signaling, there remains controversy regarding whether hypoxia increases or decreases reactive oxygen species and/or hydrogen peroxide [109].

ASIC1a has been shown to contribute to acute hypoxic pulmonary vasoconstriction, as pharmacological inhibition or genetic deletion of ASIC1a attenuates hypoxia-induced increases in pulmonary vascular resistance [97]. The molecular link between acute hypoxia and ASIC1a activation is still incompletely defined (Fig. 2). However, ASIC1a is well recognized as a redox-sensitive ion channel. Consistent with this, reducing agents increase, whereas oxidizing agents decrease, ASIC1a surface expression and acid-evoked currents [110–113]. These findings suggest that changes in cellular redox state may contribute to ASIC1a activation during acute hypoxic stress. Further studies are required to determine the specific molecular pathways linking reductions in oxygen availability to ASIC1a activation and downstream signaling.

While acute hypoxic pulmonary vasoconstriction is an adaptive response that preserves oxygenation, sustained chronic hypoxia drives vascular remodeling and contributes to pulmonary hypertension. These findings suggest that ASIC1a contributes to acute hypoxic pulmonary vasoconstriction and that dysregulation of ASIC1a-dependent signaling may promote the transition from adaptive vasoconstriction to pathological vascular remodeling.

### Chronic hypoxia-induced ASIC1a activation

**Fig. 3:**
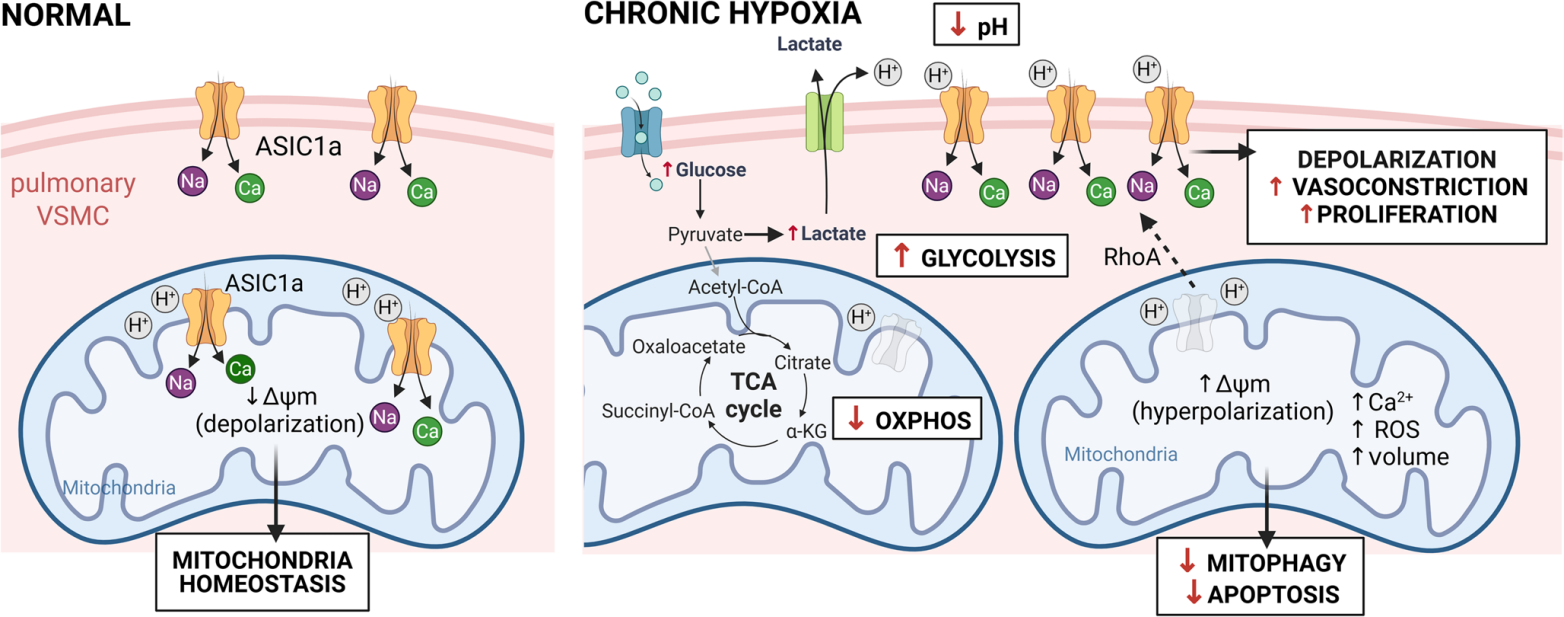
Redistribution and Activation of ASIC1a in Pulmonary Hypertension. Under normal physiological conditions, ASIC1a is relatively quiescent at the plasma membrane of pulmonary VSMCs, while mitochondrial-localized ASIC1a supports mitochondrial homeostasis. Chronic hypoxia induces a redistribution of ASIC1a characterized by increased plasma membrane localization and reduced mitochondrial expression. Increased RhoA activity following chronic hypoxia promotes plasma membrane ASIC1a expression. Loss of mitochondria ASIC1a promotes mitochondrial membrane potential (ΔΨm) hyperpolarization, accumulation of mitochondrial Ca^2+^ and reactive oxygen species (ROS), increased mitochondrial volume, impaired mitophagy, and reduced susceptibility to apoptosis. In parallel, chronic hypoxia drives a metabolic shift from oxidative phosphorylation (OXPHOS) toward aerobic glycolysis and lactate production. Increased metabolic acid generation is counteracted by activation of membrane H^+^ transporters, producing intracellular alkalization and extracellular acidification. The resulting drop in extracellular pH activates plasma membrane ASIC1a, promoting Na^+^ and Ca^2+^ influx, leading to sustained membrane depolarization, enhanced vasoconstriction, and hyperproliferation of VSMCs. Together, these compartment-specific effects of ASIC1a contribute to pulmonary vascular remodeling, increased pulmonary vascular resistance, and the development of pulmonary hypertension. Figure created with BioRender.com

In pulmonary hypertension, VSMCs undergo a metabolic shift from oxidative phosphorylation toward aerobic glycolysis and lactate production, resembling the Warburg-like phenotype often observed in cancer [114]. This metabolic reprogramming promotes a hyperproliferative, apoptosis-resistant state. This metabolic shift creates a microenvironment characterized by intracellular alkalinization and extracellular acidification, a process referred to as pH inversion [114–116]. This extracellular acidosis is a key trigger for ASIC1a activation, as inhibition of glycolysis prevents both acidification and ASIC1a-mediated store-operated Ca^2+^ entry [116] (Fig. 3). However, inhibition of glycolysis does not prevent pharmacologic activation of ASIC1a by α/β-MitTx, which produced greater ASIC1a activation in VSMCs from pulmonary hypertensive animals. This is consistent with increased surface expression following chronic hypoxia [108], indicating channel trafficking and functional activation are independently regulated.

Despite enhanced activity in pulmonary hypertension, ASIC1a gene and protein expression are not significantly altered [97, 108]. Instead, chronic hypoxia primarily increases ASIC1a function by enhancing its trafficking to the plasma membrane [108]. Several mechanisms have been implicated in regulating ASIC1a surface expression. For example, glycosylation at N393 promotes plasma membrane localization [117, 118], however the extent of ASIC1a N-linked glycosylation during chronic hypoxia remains unknown.

ASIC1 also interacts with the PDZ domain-containing protein PICK1 (protein interacting with C kinase 1), which regulates channel localization and signaling in multiple cell types [119–121]. Although PICK1 inhibition does not alter ASIC1 surface expression in pulmonary VSMCs [75], it is required for modulation of ASIC1 by PKA, PKC, and calcineurin signaling pathways [75]. In pulmonary VSMCs, ASIC1a-mediated store-operated Ca^2+^ entry is enhanced by PKA-mediated phosphorylation but inhibited by PKC- and calcineurin-dependent mechanisms, suggesting that PICK1 functions as a scaffold coordinating kinase- and phosphatase-dependent regulation of channel activity [75]. Upon store depletion, the association between ASIC1 and PICK1 increases, facilitating calcineurin-dependent dephosphorylation of ASIC1a and limiting store-operated Ca^2+^ entry (Fig. 2). ASIC1-PICK1 coupling is also required for chronic hypoxia-induced activation of the Ca^2+^-sensitive nuclear factor of activated T cells isoform c3 (NFATc3) transcription factor, thereby linking ASIC1a to downstream transcriptional remodeling [122]. Together, these findings identify PICK1 as a regulatory hub integrating phosphorylation-dependent signaling and feedback control of ASIC1a activity in pulmonary VSMCs.

Additional regulation involves annexin II light chain p11 (also known as S100A10), which has been implicated in ASIC1 trafficking and is associated with recruitment of small GTPases such as RhoA to the actin cytoskeleton [123–126]. Chronic hypoxia increases RhoA activity in pulmonary VSMCs, and inhibition of RhoA reduces ASIC1 plasma membrane expression, acid-induced Ca^2+^ influx, and store-operated Ca^2+^ entry, whereas activating RhoA has the opposite effect [108]. These observations are consistent with RhoA-dependent trafficking mechanisms described for related ENaC channels [127–129]. However, because ENaC regulation by RhoA requires an intact cytoskeleton [130], it remains unclear whether RhoA directly regulates ASIC1a or acts indirectly through cytoskeletal remodeling.

More recently, prenylcysteine oxidase 1 like (PCYOX1L) has been identified as a secreted protein that promotes ASIC1a assembly and membrane expression [131]. Whether this is a potential mechanism by which chronic hypoxia increases ASIC1a surface expression remains to be determined. Together, these findings indicate that ASIC1a activation in pulmonary VSMCs is governed by the coordinated regulation of extracellular pH, intracellular signaling, and, most importantly, the dynamic control of channel trafficking.

### Mitochondria localization and function of ASIC1a

Recent work has identified a mitochondrial pool of ASIC1a (mtASIC1a), including its detection in highly purified mitochondrial fractions, suggesting a direct role for ASIC1a in mitochondrial homeostasis [132–134]. mtASIC1a is thought to localize to the inner mitochondrial membrane, where it contributes to basal cation conductance by eliciting mild depolarizing currents in the highly negative electrochemical potential. Through this activity, mtASIC1a appears to regulate mitochondrial membrane potential and bioenergetic stability. Supporting this concept, neuronal studies demonstrate that hydrogen peroxide-induced activation of mtASIC1a promotes mitochondrial-dependent cell death [134]. These findings are particularly intriguing because hydrogen peroxide largely inhibits plasma membrane ASIC1a activity in pulmonary VSMCs [112], suggesting that distinct subcellular pools of ASIC1a may be differentially regulated through compartment-specific trafficking, redox sensitivity, or post-translational modification.

Chronic hypoxia causes a redistribution of ASIC1a, characterized by increased plasma membrane expression accompanied by a marked reduction in mitochondrial localization [133]. Loss of mtASIC1a, either through chronic hypoxia or genetic deletion, results in hyperpolarization of the mitochondrial membrane potential. This shift promotes enhanced mitochondrial Ca^2+^ uptake, increased reactive oxygen species production, and disruption of mitochondrial quality-control mechanisms like mitophagy (Fig. 3). Additionally, loss of mtASIC1a led to mitochondrial morphological defects, including larger size, decreased aspect ratio, and reduced number, indicating mitochondrial enlargement, although expression of major fusion/fission proteins is largely unchanged [133].

Functionally, loss of mtASIC1a in VSMCs is associated with cell survival and low susceptibility to apoptosis. Of note, selective restoration of mtASIC1a is sufficient to normalize mitochondrial membrane potential, reduce mitochondrial reactive oxygen species, and rescue caspase-3 activation [133], demonstrating a direct and compartment-specific role for ASIC1a in mitochondrial regulation.

Together, these data suggest mtASIC1a contributes to the regulation of mitochondrial homeostasis in pulmonary VSMCs and identify ASIC1a as a dual-function ion channel with distinct, but complementary, compartment-specific roles. Whereas plasma membrane ASIC1a modulates Ca^2+^-dependent signaling, mtASIC1a influences bioenergetics, redox balance, and apoptotic sensitivity. Dynamic regulation of ASIC1a subcellular localization, therefore, represents an important mechanism by which cells integrate extracellular and intracellular stress signals to coordinate function outcomes.

### ASICs in pulmonary hypertension

Pulmonary hypertension is a progressive vascular disease characterized by sustained vasoconstriction, vascular remodeling, and increased pulmonary arterial pressure, eventually resulting in right ventricular failure. These pathological changes arise from multiple interacting processes, including metabolic reprogramming, smooth muscle hyperreactivity, endothelial dysfunction, excessive proliferation, and impaired apoptosis [135–137]. A central feature underlying these events is dysregulated ion channel activity, which promotes VSMC membrane depolarization and elevated [Ca^2+^]_*i*_.

**Fig. 4:**
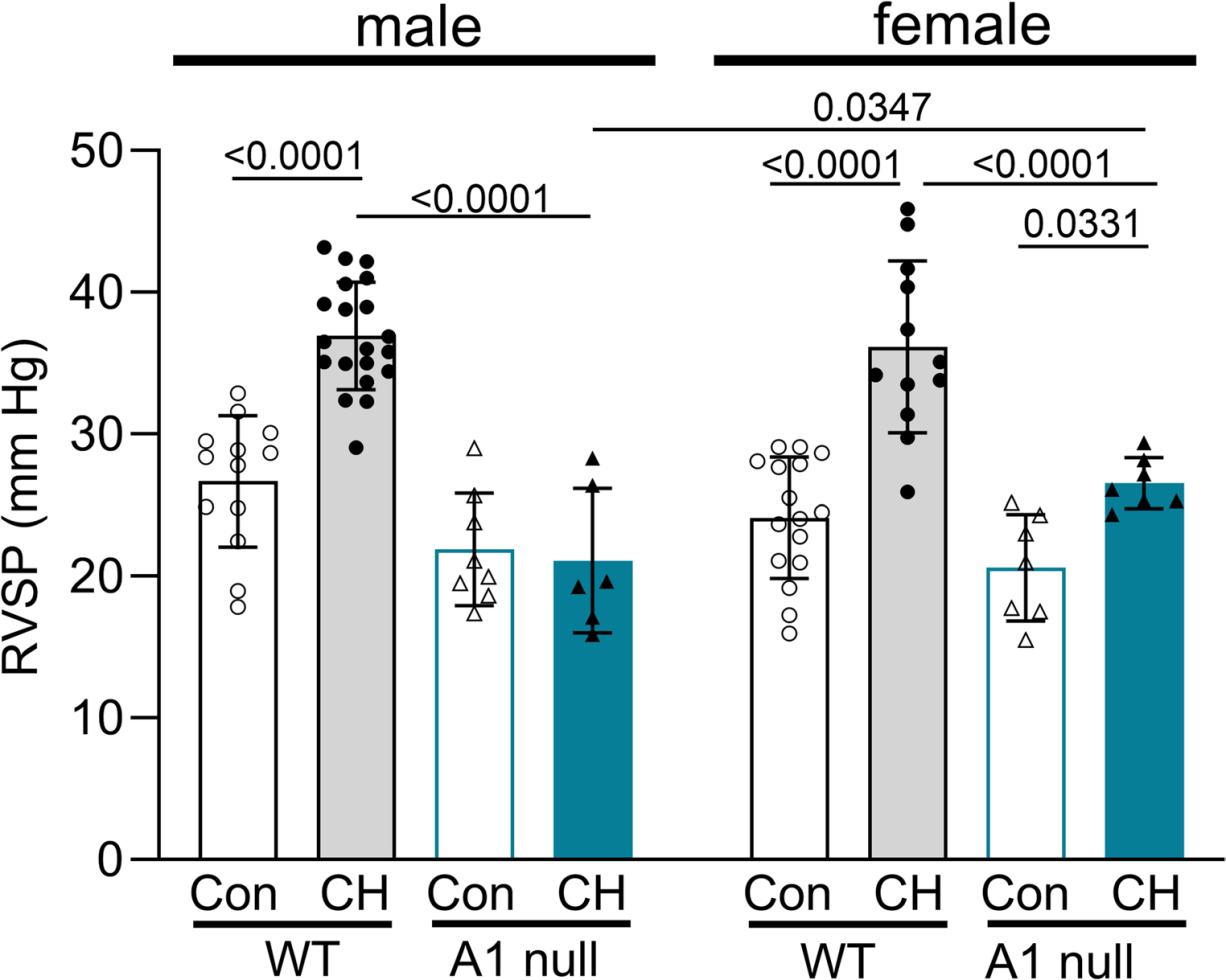
Sex-dependent contribution of ASIC1a to chronic hypoxia-induced pulmonary hypertension. Right ventricular systolic pressure (RVSP, mm Hg), an index of pulmonary arterial pressure, was measured in male and female wild-type and ASIC1a-null mice under control (Con) conditions or following 4 weeks of chronic hypoxia (CH; 0.5 atm). Chronic hypoxia produces comparable increases in RVSP in male and female wild-type mice. Deletion of ASIC1a abolishes the hypoxia-induced increase in RVSP in males, whereas female ASIC1a-null mice retain a modest but significant elevation in RVSP. Data are mean ± SD (*n* = 6–20 animals/group). Previously pooled male and female datasets were separated and reanalyzed by two-way ANOVA with Tukey’s multiple comparison test [3, 97].

Consistent with the role of ASICs in pulmonary vascular regulation, genetic studies show divergent contributions of ASIC isoforms to disease progression. ASIC1a deficiency protects against the development of chronic hypoxia-induced pulmonary hypertension [3, 97], whereas loss of ASIC2 exacerbates pulmonary vascular remodeling and increases pulmonary arterial pressure, and deletion of ASIC3 has little effect on disease development [56]. Importantly, smooth muscle-specific deletion of ASIC1a prevents the development of pulmonary hypertension and can reverse established disease, whereas endothelial-specific deletion has minimal effects on disease severity, but may worsen vascular remodeling [3]. While early studies combined male and female animals [56, 97], subsequent work suggests sex-dependent differences in the contribution of ASIC1a to pulmonary hypertension [3]. Although estrogen has been reported to protect female rats in experimental models of pulmonary hypertension [138–140], chronic hypoxia produces comparable increases in right ventricular systolic pressures in wild-type male and female mice (Fig. 4). Notably, deletion of ASIC1a completely prevents this response in males, whereas female ASIC1a-null mice exhibit a modest but significant elevation in right ventricular systolic pressure, suggesting female mice rely on ASIC1a-independent mechanisms to develop hypoxia-induced pulmonary hypertension. What drives this sex-specific difference remains an important area of further investigation.

A hallmark of pulmonary hypertension is persistent depolarization of the VSMC membrane potential, driven in part by reduced K^+^ channel expression and activity [141–146]. Under these conditions, decreased K^+^ permeability increases the relative contribution of inward cation currents. Although ASIC1a does not affect membrane potential under control conditions, ASIC1a-dependent Na^+^ influx contributes to the chronic hypoxia-mediated depolarization, despite persistent suppression of K^+^ currents [147]. These data suggest that increased ASIC1a activity, in concert with reduced K^+^ conductance, is important for maintaining the depolarized state of pulmonary VSMCs.

Although membrane depolarization can activate voltage-gated Ca^2+^ channels [98, 148, 149], Ca^2+^ entry in distal pulmonary arteries is only partially dependent on these channels. Instead, non-voltage-gated pathways, including store-operated and receptor-operated Ca^2+^ entry, predominate [98, 148, 150, 151]. ASIC1a contributes to these pathways [2, 72, 98], amplifying Ca^2+^ influx that drives not only vasoconstriction but also key remodeling processes, including VSMC proliferation, migration, and resistance to apoptosis [3, 133]. In addition, ASIC1a-dependent Ca^2+^ influx activates the Ca^2+^-sensitive transcription factor NFATc3, which promotes gene programs that sustain the pathological phenotype [122].

In parallel, the loss of the mitochondrial pool of ASIC1a in pulmonary hypertension introduces an additional layer of regulation that favors VSMC survival by disrupting redox balance, impairing mitophagy, and reducing apoptotic susceptibility (Fig. 3). Together, these compartment-specific functions of ASIC1a act as a dual regulator of cytosolic signaling and mitochondrial homeostasis, with dysregulation of both pools contributing to pulmonary vascular disease.

## Therapeutic potential and challenges

A comprehensive overview of ASIC inhibitors and activators has been detailed elsewhere [1]. Although ASICs represent a promising therapeutic target in vascular disease, their diverse function across cell types and subcellular compartments presents significant challenges. For instance, ASIC1a-mediated Na^+^ and Ca^2+^ influx regulates endothelium-dependent hyperpolarization and vasodilation, as well as smooth muscle depolarization and contraction. Moreover, ASIC1a exhibits compartment-specific signaling, with distinct roles at the plasma membrane versus within mitochondria.

The most selective modulators of ASIC1a are the peptide inhibitors derived from spider venom, such as psalmotoxin 1 and Hi1a (Hadronyche infensa venom peptide 1a), and the potent peptide activator, α/β-MitTx, which is derived from the venom of the Texas coral snake. While these peptides exhibit high specificity and efficacy for ASIC1a in preclinical models, their clinical use remains limited. The relatively large size and hydrophilic nature of the peptides restrict membrane permeability. This can limit access to ASIC1a within the vascular wall as well as ASIC1a localized in the mitochondria. In addition, peptides are rapidly degraded by proteases, resulting in short half-lives. This often necessitates frequent dosing to maintain effective concentrations. Most peptides are not orally bioavailable and require parenteral administration. These repeated exposures can trigger immunogenicity or loss of activity due to structural instability.

Recent drug discovery efforts have also identified small-molecule antagonists of ASIC1a with improved pharmacological properties relative to peptide toxins. Compound 5b (C5b), a small molecule designed based on the ASIC1a-PcTx1 binding interface, potently and selectively inhibits ASIC1a-containing channels and, unlike peptide toxins, is blood-brain barrier permeable and effective following systemic intravenous administration, significantly reducing infarct volume in a mouse model of ischemic stroke [152]. While developed and validated in the context of cerebral ischemia, this class of small-molecule, structure-based ASIC1a antagonists may offer a more translationally viable scaffold for future development of vascular-targeted ASIC1a inhibitors, circumventing the delivery and stability limitations inherent to a peptide-based approaches. JNJ-799,760 and JNJ-67,869,386 are two other small-molecule antagonists with a distinct mechanism of action. Both are potent, voltage-independent allosteric antagonists that bind to a novel site within the acidic pocket of ASIC1a, stabilizing the channel’s closed state and slowing its activation kinetics, in contrast to the pore-blocking mechanism of other inhibitors such as amiloride [153, 154]. While these compounds have been well characterized structurally and mechanistically, their efficacy and pharmacokinetic properties in vivo have not yet been reported. Notably, amiloride remains the only ASIC inhibitor in clinical use; however, its poor subtype selectivity and broader inhibition of other ion channels increase the risk of off-target effects, limiting its utility as a selective ASIC1a-targeted therapy.

A potentially more successful strategy is to target ASIC1a trafficking and subcellular localization rather than global channel inhibition. Given that disease progression is associated with altered compartmentalization, modulating the signaling pathways and protein interactions that govern channel trafficking may allow selective suppression of pathological signaling whilst preserving physiological function. Such an approach also raises the possibility of compartment-specific targeting, enabling differential regulation of plasma membrane versus mitochondrial ASIC1a pools. As discussed above, RhoA regulates ASIC1a plasma membrane trafficking in PASMC [108], and pharmacological inhibition of RhoA reduces ASIC1a-mediated Ca^2+^ influx. Because RhoA activation depends on isoprenylation, a process inhibited by statins, which are routinely used in the clinic as cholesterol-lowering drugs, these existing RhoA-modulating agents could potentially be repurposed to target ASIC1a trafficking, representing a translationally accessible strategy warranting further investigation.

## Gaps in knowledge and future directions

Despite substantial progress, several major gaps remain in our understanding of vascular ASIC signaling. While much of the field has focused on ASIC1a, the broader ASIC family remains poorly characterized in the vasculature. In particular, the role of ASIC4 is largely unknown. Unlike other family members, ASIC4 does not form functional homomeric channels, yet it is expressed in vascular-relevant tissues and may exert modulatory effects through heteromeric assembly with ASIC1a or other subunits [7, 22, 54]. The contribution of heteromeric ASIC channels, more broadly, including their subunit composition, biophysical properties, and sensitivity to physiological and pathological stimuli, remains a significant gap in the vascular field that has received little attention. Defining the full complement of ASIC subunits expressed across vascular beds and how heteromeric assembly shapes channel function will be an important foundation for understanding vascular ASIC biology.

Building on this, a central unresolved issue is how ASIC1a is differentially activated across vascular beds, cellular contexts, sexes, and disease states. Although extracellular acidosis is a well-established stimulus, accumulating evidence supports non-proton mechanisms involving receptor signaling, kinase pathways, redox modulation, and altered channel trafficking. An important unresolved question is how ASIC1a signaling becomes selectively coupled to receptor-operated versus store-operated pathways in different vascular cell types. For example, mesenteric endothelial cells primarily engage receptor-operated, PKC-dependent ASIC1a signaling, whereas pulmonary VSMCs preferentially engage store-operated mechanisms linked to STIM1 coupling [2, 75]. Defining how these distinct signaling modalities operate across vascular beds will be critical for understanding the physiological and pathological functions of ASIC1a in the vasculature. How physiological context and vascular bed identity more broadly shape ASIC function and output is explored further in the Perspectives section below.

Equally important is the role of ASICs in extra-vascular cells within the vascular niche. Most research has focused on smooth muscle and endothelial cells, yet immune cells, fibroblasts, pericytes, and vascular stem and progenitor cells also reside in close proximity to the vessel wall and are increasingly recognized as active regulators of vascular tone, remodeling, and disease. Whether and how ASIC signaling in these cell populations contributes to vascular function remains largely unknown and represents a compelling area for future investigation.

Sex differences in vascular ASIC signaling also warrant greater attention. Emerging evidence from ASIC1a-null mice reveals a striking sexual dimorphism in cardiovascular function. In the systemic circulation, ASIC1a protects male but not female mice from age-dependent hypertension [47]. Whether these sex-dependent differences reflect direct hormonal regulation of ASIC1a by estradiol [48–50], or indirect effects mediated through sex-dependent neuroendocrine pathways remains unknown. In the pulmonary circulation, ASIC1a deletion, including cell type-specific deletion in VSMC, completely prevents pulmonary hypertension in male mice, but female null mice retain a modest but significant elevation in right ventricular systolic pressure. This suggests that female mice engage ASIC1a-independent mechanisms to develop pulmonary hypertension, the identity of which remains an important open question. More broadly, the influence of biological sex on ASIC expression, regulation, and function across vascular beds and disease contexts is poorly defined and deserves a dedicated study.

An additional gap is understanding the processes regulating ASIC1a trafficking and compartmentalization. The signals that direct ASIC1a to the plasma membrane versus mitochondrial pools, and how these processes are dynamically regulated during physiological versus pathological states, remain poorly defined. Elucidating these pathways will be essential, particularly given emerging evidence that mitochondrial dysfunction is associated with disease progression. The functional interplay between the plasma membrane and mitochondrial ASIC1a also remains unclear; whether these pools operate independently or are coordinated within an integrated signaling network remains unknown, and answering these questions will be important for understanding how to therapeutically target ASIC1a.

Most current knowledge is derived from animal models, and the relevance of these findings to human vascular disease requires further validation. Therapeutic development is still in its early stages. Future efforts should focus on identifying selective modulators of ASIC1a function, improving delivery strategies for peptide-based inhibitors, and targeting the molecular machinery that regulates ASIC1a localization and subunit assembly. Approaches that enable compartment-specific or cell-type-specific modulation may offer particular promise, allowing selective targeting of pathological signaling while preserving normal vascular function. Addressing these gaps will be essential for fully defining the role of ASICs in vascular biology and for translating these insights into effective therapies for vascular disease.

## Perspectives

An emerging theme from the study of vascular ASICs is that physiological context fundamentally shapes channel function. Individual vascular beds are functionally specialized to meet distinct local demands, and as a result, the same stimulus can elicit opposing responses depending on the vascular bed. Hypoxia illustrates this concept clearly: in the pulmonary circulation, hypoxia elicits vasoconstriction, a homeostatic mechanism that redirects blood flow away from poorly ventilated lung regions to optimize ventilation-perfusion matching, whereas in the systemic circulation, hypoxia elicits vasodilation, which increases oxygen delivery to metabolically active or ischemic tissues. This principle has profound implications for how we interpret ASIC function across the vasculature. ASIC1a contributes to the hypoxic pulmonary vasoconstrictor response [54]. Whether ASIC1a participates in systemic hypoxic-induced vasodilation is unknown, though its role in endothelial-mediated dilation suggests it may. If so, ASIC1a would serve opposing vasoactive roles depending on the circulation, underscoring how profoundly vascular bed identity can shape the functional output of the same channel.

This framework extends beyond hypoxia to include acidosis, mechanical stimuli, and neuroendocrine signals, all of which interact with the vasculature in bed-specific ways, and ASIC responses to these stimuli are likely shaped accordingly. The emerging evidence for sex-dependent differences in ASIC1a function across both the pulmonary and systemic circulations adds an additional complexity to an already heterogeneous system.

Ultimately, vascular bed identity should be viewed as a core variable in ASIC biology, providing a fundamental organizing principle that shapes how these channels are regulated, what signals they respond to, and what functional outcomes their activation produces. While these considerations have direct therapeutic implications, embracing this complexity will be essential for moving the field forward and for translating basic discoveries in ASIC biology into targeted, context-dependent therapeutic strategies.

## Data Availability

The data included in Figure 4 were from previously published studies in which male and female data had originally been pooled [33, 67]. These datasets were subsequently separated by sex and reanalyzed.
